# Single-incision laparoscopic splenectomy with innovative gastric traction suture

**DOI:** 10.4103/0972-9941.72386

**Published:** 2011

**Authors:** G Srikanth, M D Wasim, A Sajjad, Neel Shetty

**Affiliations:** Department of Surgical Gastroenterology, Manipal Institute of Liver and Digestive Diseases, Manipal Hospital, Bangalore, India

**Keywords:** Gastric traction suture, ITP, single-incision laparoscopic splenectomy

## Abstract

Laparoscopic splenectomy is now the gold standard for patients with idiopathic thrombocytopenic purpura (ITP) undergoing splenectomy. There are a few reports in literature on single-incision laparoscopic (SIL) splenectomy. Herein, we describe a patient undergoing SIL splenectomy for ITP without the use of a disposable port device. We report a 20-year-old female patient with steroid-refractory ITP having a platelet count of 14,000/cmm who underwent a SIL splenectomy. Dissection was facilitated by the use of a single articulating grasper and a gastric traction suture and splenic vessels were secured at the hilum with an endo-GIA stapler. She made an uneventful postoperative recovery and was discharged on the second postoperative day. She is doing well with no visible scar at 8-month follow-up.

## INTRODUCTION

Laparoscopic splenectomy is now the gold standard for patients with idiopathic thrombocytopenic purpura (ITP) undergoing splenectomy. A variety of single-incision laparoscopic (SIL) procedures have been described in the last two years, viz; cholecystectomy, hernia repair, appendectomy, gynecological procedures, nephrectomy, donor nephrectomy, pyeloplasty, right hemicolectomy, sigmoidectomy, gastric banding and sleeve gastrectomy.[1–5] Herein, we report a patient who underwent SIL splenectomy for steroid-refractory ITP.

## CASE REPORT

**Figure 1:**
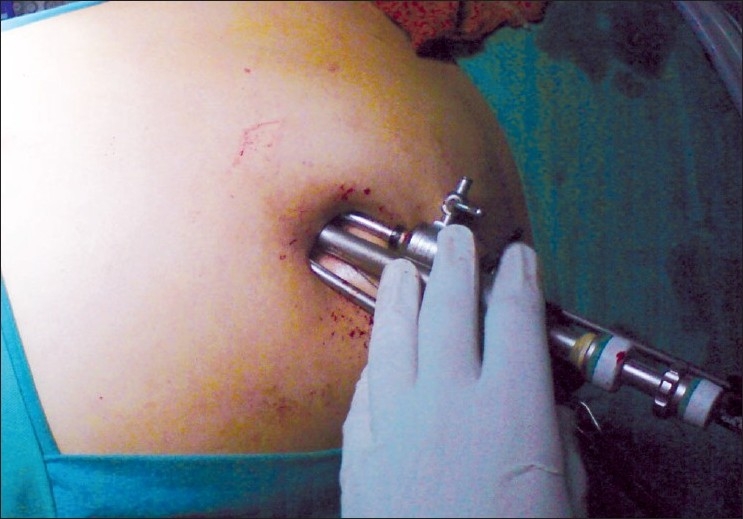
One 10 mm and two 5 mm ports placed through single-incision trans-umblically

**Figure 2:**
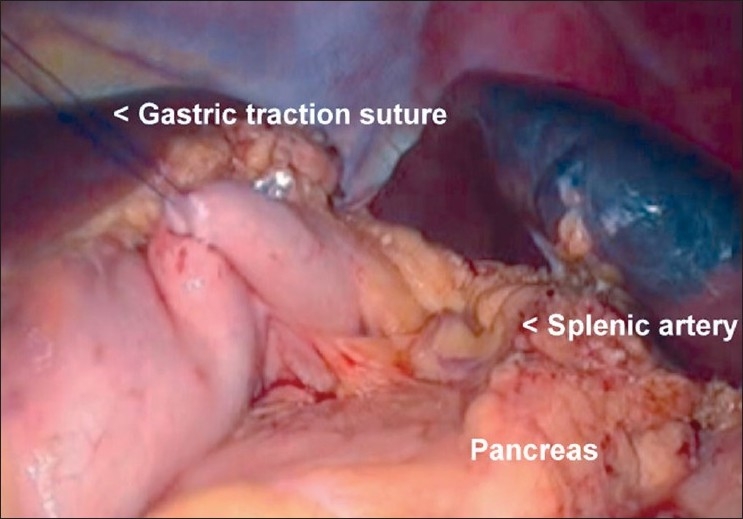
Gastric traction suture in place providing wide exposure of lesser sac

**Figure 3:**
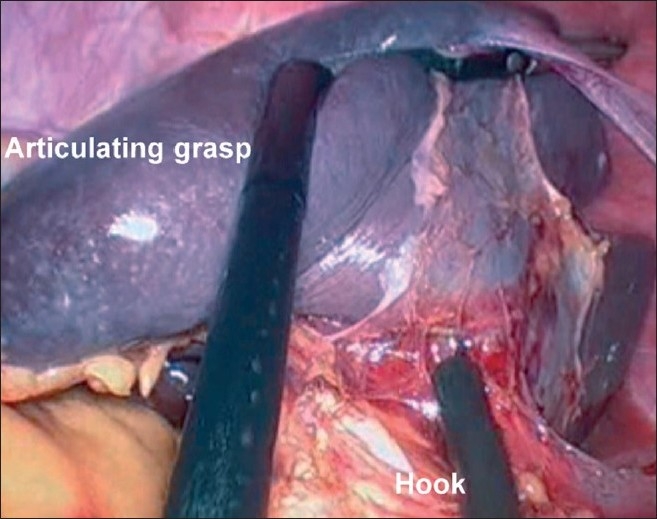
Articulating grasper retracting the lower pole of the spleen anteriorly, exposing the posterior splenic attachments

**Figure 4:**
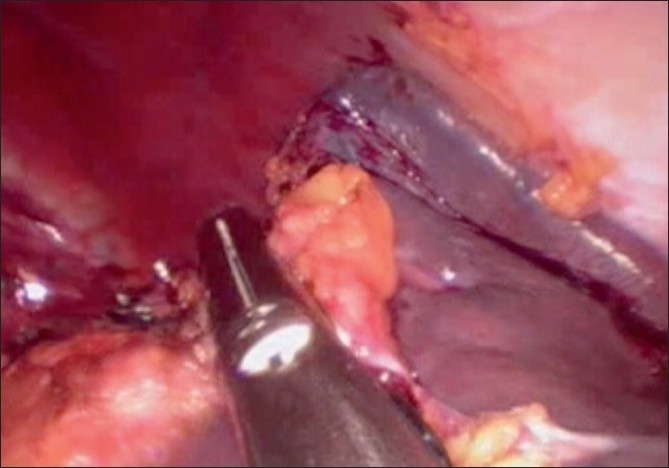
Endo GIA staple transaction of splenic hilum.

**Figure 5:**
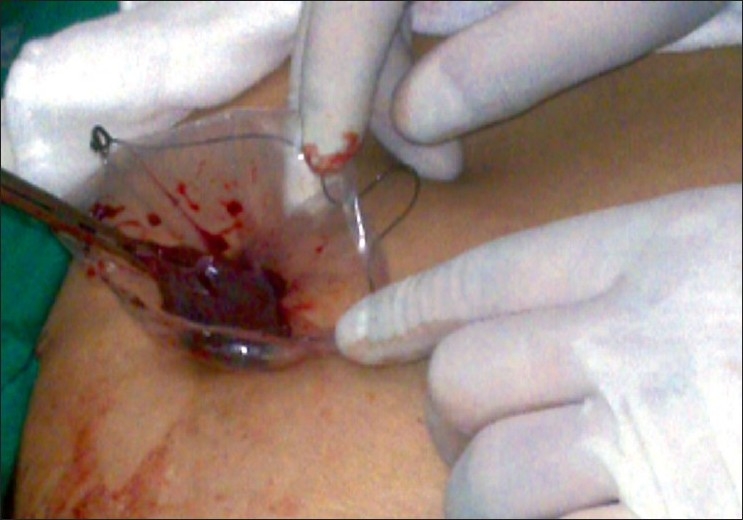
Morselled spleen being retrieved transumblically in an endobag.

A 20-year-old female patient with steroid refractory ITP was referred for splenectomy. Her platelet count was 14,000/cmm preoperatively. She received pneumococcal and meningococcal vaccines 2 weeks prior to surgery. The patient received prophylactic antibiotic before surgery. She underwent SIL splenectomy. Surgery was performed under general endotracheal anaesthesia with the patient in the right lateral position. Firstly, the umbilicus was everted using a Littlewoods forceps. A skin incision of 20 mm was made in right hemi-circumference of umbilicus. Care was taken to keep the incision within the umbilical ring for the best cosmetic outcome. A mixture of sharp and blunt dissection was used down to the linea alba. The sheath was cleared for a distance of 1 cm around the umbilical ring. The sheath and peritoneum were opened under direct vision, and a 10-mm laparoscopic port inserted. Pneumoperitoneum was then established. Two 5-mm ports were then inserted through the fascia on either side of the 10-mm port. All the ports were ones used in conventional laparoscopic surgery [Figure 1]. The spleen was of normal size. The splenic flexure of the colon was mobilised and the lesser sac was entered. The stomach was retracted by an innovative gastric traction suture [Figure 2]. A 2-0 polypropyelene suture was used to place a seromuscular stitch on the posterior wall of stomach and the ends of this suture were exteriorised in the epigastrium using the suture retrieval method described by Mattie.[6] External traction on the exteriorised suture resulted in gastric retraction cranially and to the right, thus widely exposing the lesser sac. Short gastric vessels were then divided with harmonic shears (Ethicon Endosurgery, Mumbai, India). Lower pole attachments of spleen and lienorenal ligament were divided with monopolar hook cautery. Dissection was facilitated by the use of single articulating grasper in one hand and regular laparoscopic instruments in the other [Figure 3]. The 10-mm port was then exchanged with a 12-mm port and splenic vessels were secured at hilum with an endo-GIA stapler (Autosuture, 45 mm White cartridge, 2.5 mm stapler length) [Figure 4]. The three port sites were connected and the morselled spleen was retrieved within an endobag through the umbilical incision [Figure 5]. Meticulous closure of the sheath was performed with 1-0 absorbable suture. Skin closure was done with glue.

**Figure 6:**
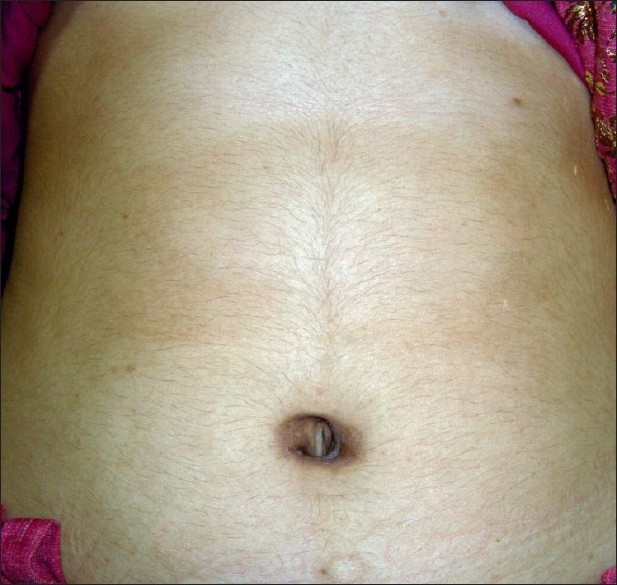
Scar at 8 months follow-up after surgery

The operative time was 150 minutes. The postoperative period was uneventful and she was discharged on the second postoperative day on full diet. At follow up in the clinic at 1 week and 1 month the scar was not discernible. At 8 months follow-up there was no umbilical hernia and the cosmetic result was excellent [Figure 6].

## DISCUSSION

SIL surgery is a new frontier in laparoscopic surgery. Feasibility of several basic and advanced laparoscopic surgeries has been demonstrated. Barbaros *et al*., and Targarano *et al*., have recently reported on transumblical SIL splenectomy in patients with ITP.[1,3]

SIL approach for solid organs poses several technical challenges apart from the instrument conflicts, difficulty in visualisation and range of movements. Firstly, solid organs cannot be grasped and retracted. Secondly, during splenectomy, exposure may be suboptimal because of inadequate exposure of the lesser sac and hilum of spleen. Thirdly, release of posterior attachments can be technically difficult because robust anterior retraction of spleen may be difficult to achieve. Fourthly, bagging the specimen and extraction can be challenging. To overcome these challenges, Targarona *et al*, have described the use of a flexible endoscope, trans-anal endoscopic instruments and in two cases utilisation of a subcostal single-incision as access site for the SIL splenectomy.[4]

The lateral position of the patient has been reported in all the reported cases of SIL splenectomy in the literature.[2–5] We found that this position provided an excellent view of the operative field. In the author’s experience (GS) of more than 100 different SILS procedures, avoidance of disposable port would give advantages of better ergonomics, a greater range of movements of instruments than the available port devices and has an added cost benefit. In our patient, dissection was performed with regular laparoscopic instruments and a single articulating grasper for facilitating retraction. The articulating grasper may help in better counter traction and visualisation, as well as avoids instrument conflicts during surgery. The long 45° laparoscope was used for vision to avoid instrument conflicts between the operating surgeon and the camera assistant. The gastric traction suture provided wider exposure of lesser sac, facilitated division of short gastric vessels, dissection of the splenic hilum and excellent view of the splenic bed after splenectomy for checking hemostasis. In our case, we could place the spleen in an endobag, morcellate it and retrieve it comfortabley. However, we feel that this could pose a significant technical challenge in a larger spleen.

In conclusion, SIL splenectomy is feasible without a disposable port device. Apart from excellent cosmesis, it has the possible advantages of reduced postoperative pain and faster return to normal activity. Larger studies are required for comparing SIL splenectomy with conventional laparoscopic splenectomy to address indications, technical challenges and comparing two approaches.
